# A lifestyle-driven inflammatory load index and its circulating metabolomic signature identify susceptibility to hospital-recorded acute respiratory infections: a population-based cohort study of 295,679 adults

**DOI:** 10.3389/fendo.2026.1878728

**Published:** 2026-08-10

**Authors:** Xufeng Fu, Xiaohua Li, Xinqian Ma, Nan Wu, Wentao Ni, Hui Li

**Affiliations:** 1 Department of Respiratory and Critical Care Medicine, The Seventh Affiliated Hospital of Sun Yat-sen University, Shenzhen, China; 2 Department of Respiratory and Critical Care Medicine, Fujian Medical University Affiliated Fuzhou First Hospital, Fuzhou, China; 3 Department of Respiratory and Critical Care Medicine, Human Genome Research Center, Peking University People’s Hospital, Beijing, China

**Keywords:** acute respiratory infection, biomarkers, immunometabolism, inflammatory load index, lifestyle factors, low-grade systemic inflammation, metabolomic signature

## Abstract

**Background:**

Whether a population-scale lifestyle-anchored inflammatory load index and its circulating metabolomic risk signature identify susceptibility to hospital-recorded acute respiratory infections (ARIs) has not been well established. This study examined these questions in the UK Biobank using the Circadian Imbalance Index (CII) and a nuclear magnetic resonance (NMR)–derived metabolomic risk signature (MRS).

**Methods:**

This prospective cohort study included 295,679 UK Biobank participants after prespecified exclusions. The CII was derived from five baseline components: evening chronotype, atypical sleep duration, high neuroticism, atypical coffee intake, and low vitamin D, and was categorized as low (0-1), intermediate (2-3), or high (4-5). Cox proportional-hazards models evaluated associations between CII and incident hospital-recorded ARIs. Among 264,889 participants with complete baseline Nightingale NMR metabolomics, an MRS was derived from 249 standardized biomarkers by regressing standardized CII on the biomarker panel using elastic net regularized regression. Counterfactual mediation models estimated natural direct and indirect associations for lower respiratory tract infection (LRTI) and pneumonia.

**Results:**

Higher CII was associated with greater risk across hospital-recorded ARI outcomes. In the fully adjusted model, high versus low CII was associated with higher risks of LRTI (hazard ratio [HR], 1.56; 95% confidence interval [CI], 1.49 to 1.63) and pneumonia (HR, 1.56; 95% CI, 1.48 to 1.64). Ten-year cumulative incidence of LRTI increased from 3.87% in the low CII group to 6.56% in the high CII group; corresponding pneumonia estimates were 2.69% and 4.52%. The MRS accounted for 18.5% (95% CI, 16.2% to 20.7%) of the continuous-CII association with LRTI and 19.0% (95% CI, 16.4% to 21.6%) of the association with pneumonia.

**Conclusion:**

In a population-scale cohort, a lifestyle-anchored inflammatory load index showed a graded association with hospital-recorded ARIs, and a circulating NMR-derived MRS, dominated by fatty-acid-related biomarkers and including the established low-grade-inflammation marker glycoprotein acetylation (GlycA), accounted for approximately one-fifth of the association with LRTI.

## 1 Introduction

Chronic, low-grade systemic inflammation has emerged as a unifying pathophysiological substrate underlying a wide spectrum of chronic inflammatory diseases, including cardiometabolic, autoimmune, neurodegenerative, and respiratory conditions (1). Modifiable lifestyle factors, including sleep, psychosocial stress, dietary patterns, physical activity, and hormonal status, collectively shape this inflammatory milieu through immunometabolic, hormonal, and microenvironmental pathways (1, 2). Acute respiratory infections (ARIs) offer a clinically anchored setting in which to examine whether chronic low-grade inflammatory and immunometabolic vulnerability is reflected in subsequent infection susceptibility. Whether a cumulative lifestyle phenotype, integrated as a single inflammatory load index, identifies individuals at higher risk of ARIs, and whether circulating metabolomic biomarkers can capture the immunometabolic intermediates of this lifestyle-disease axis at the population scale, remain unresolved.

Existing evidence supports links between lifestyle-related exposures and immune-inflammatory regulation relevant to respiratory host defense. Sleep and circadian disruption can alter cytokine homeostasis, leukocyte trafficking, epithelial barrier integrity, antimicrobial responses, and pulmonary inflammatory responses to viral challenge (3–9). Psychosocial stress has been linked to common-cold susceptibility and immune dysregulation (10–12), vitamin D is involved in innate antimicrobial responses and has shown heterogeneous protection against ARIs in trial syntheses (13–16), and caffeine-related behavior can shift circadian phase under controlled conditions (17). These findings support the broader concept that lifestyle-driven low-grade inflammation may drive immunometabolic dysregulation, yet these domains are still most often studied as separate exposures rather than as an integrated inflammatory phenotype.

In this study, a Circadian Imbalance Index (CII), comprising chronotype, atypical sleep duration, high neuroticism, atypical coffee intake, and low vitamin D, was operationalized as a lifestyle-anchored inflammatory phenotype that integrates the sleep, psychosocial, dietary, and hormonal dimensions highlighted in the lifestyle-low-grade-inflammation framework (1, 2, 18). Circadian clock research provides one biological anchor: daily rhythms organize leukocyte trafficking, cytokine release, epithelial barrier integrity, antimicrobial responses, and pulmonary inflammatory responses to viral challenge (3–7).

The association between CII and incident hospital-recorded ARIs was examined in the UK Biobank cohort, with emphasis on lower respiratory tract infection (LRTI) and pneumonia as clinically important lower-respiratory outcomes. A circulating metabolomic risk signature (MRS) was further derived from nuclear magnetic resonance (NMR)-quantified biomarkers to serve as a candidate circulating inflammatory load index (19–23), and counterfactual mediation models (24, 25) were applied to quantify the proportion of the lifestyle-infection association mediated through the NMR-derived MRS.

## 2 Materials and methods

### 2.1 Data source and population

This prospective cohort study used data from UK Biobank, which enrolled more than 500,000 adults aged 40 to 69 years from 22 assessment centers across the United Kingdom between 2006 and 2010 (26). All participants provided written informed consent at recruitment, including consent for linkage to electronic health records and use of de-identified data for approved health-related research. Ethical approval was obtained from the North West Multi-center Research Ethics Committee (reference: 16/NW/0274). This research was conducted using the UK Biobank Resource under application number 688295. All data use and analyses were performed in strict accordance with UK Biobank’s regulations and policies.

**Figure 1:**
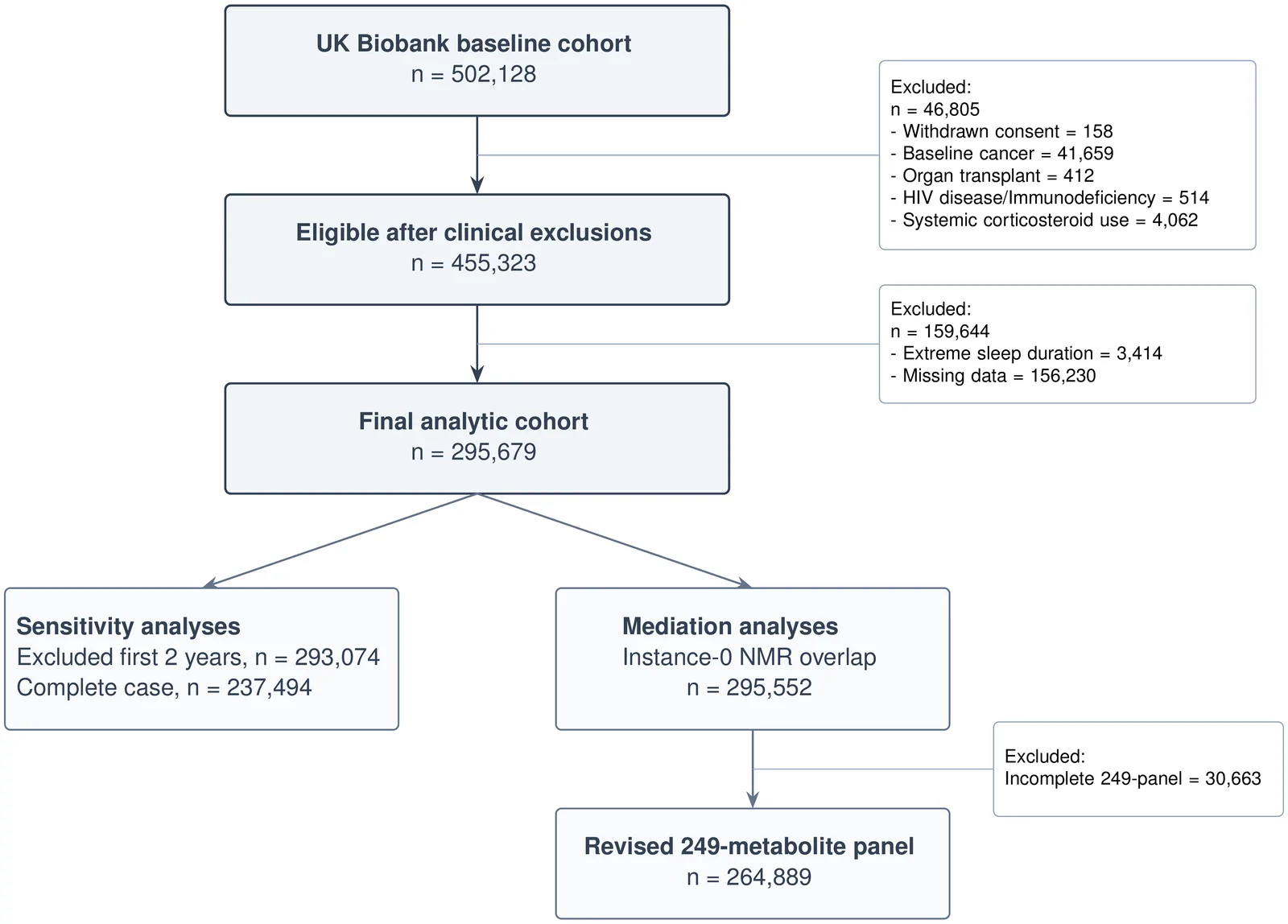
Study population flow diagram for the UK Biobank cohort. CII, Circadian Imbalance Index; UKB, UK Biobank.

Participants with withdrawn consent, baseline cancer, prior organ transplant, HIV disease/immunodeficiency, systemic corticosteroid use, extreme reported sleep duration, or missing or invalid CII component data were excluded according to the study flow shown in **Figure 1**. Follow-up was ascertained through linked hospital inpatient, death, and loss-to-follow-up records, with administrative censoring on March 31, 2023 for England, August 31, 2022 for Scotland, and May 31, 2022 for Wales.

### 2.2 Exposure assessment

The exposure was CII, constructed from five binary components: evening chronotype (field 1180), atypical sleep duration (field 1160), high neuroticism (field 20127), atypical coffee intake (field 1498), and low vitamin D (field 30890) (18). The five CII components map directly onto core lifestyle dimensions widely implicated in chronic low-grade inflammation: sleep behavior (chronotype, atypical sleep duration), psychosocial trait (high neuroticism), dietary pattern (atypical coffee intake), and hormonal status (low vitamin D, reflecting both dietary intake and endocrine signaling). Evening chronotype was defined as a response indicating a greater preference for evening than morning. Atypical sleep duration was defined as ≤6 hours or ≥9 hours per day, with 7–8 hours as the reference category. Participants reporting <3 or >12 hours of sleep were excluded prior to CII derivation. Therefore, within the analytic cohort, the atypical sleep component represented sleep durations of 3–6 hours or 9–12 hours per day. High neuroticism was defined as a neuroticism score of 7--12, with scores of 0–6 as the reference category. Atypical coffee intake was defined as no coffee intake, ≥5 cups per day, or 1–4 cups per day of decaffeinated coffee; the reference pattern was 1–4 cups per day of non-decaffeinated coffee. This heterogeneous coffee component was treated as a study-specific marker of caffeine-related timing rather than as evidence that any particular coffee consumption pattern is inherently harmful. Low vitamin D was defined as serum vitamin D level <50 nmol/L. The CII score was the sum of complete component indicators and ranged from 0 to 5. Primary association analyses categorized participants as low CII (0-1), intermediate CII (2-3), or high CII (4-5), with low CII as the reference.

### 2.3 Outcome ascertainment

Incident hospital-recorded ARIs were identified from linked hospital inpatient records using International Classification of Diseases, Tenth Revision (ICD-10) diagnosis codes. Outcomes followed a prespecified three-level hierarchy. Level 1 comprised upper respiratory tract infection (URTI; J00-J06) and LRTI (J12-J22). Level 2 separated influenza (J09-J11), pneumonia (J12-J18), and other LRTI (J20-J22). Pneumonia subtypes included viral pneumonia (J12), bacterial pneumonia (J13-J15), and unspecified pneumonia (J18).

Event dates were defined by the first post-baseline hospitalization record for the relevant ICD-10 group. Participants were followed from baseline until the first event for a given outcome, death, loss to follow-up, or administrative censoring, whichever came first.

### 2.4 Covariates

Covariates were selected *a priori* as potential confounders of the CII-respiratory infection association and grouped into three domains: sociodemographic factors (age, sex, ethnicity, Townsend deprivation index, and educational attainment), lifestyle and anthropometric factors (smoking status, alcohol frequency, body mass index (BMI), and physical activity), and clinical factors (multimorbidity category). Ethnicity was coded as White or non-white. Education was grouped as O levels or less, A levels, or college degree; smoking as current or not current; and alcohol frequency as regular or non-regular. Multimorbidity was defined from hypertension, diabetes, coronary heart disease, and stroke and categorized as 0, 1, or 2 or more conditions.

### 2.5 MRS construction

Baseline metabolomic biomarkers were measured using the Nightingale high-throughput NMR metabolomics platform (20, 27, 28). Conceptually, the MRS was constructed as a circulating, NMR-anchored biomarker score intended to capture the immunometabolic and low-grade inflammatory state associated with the lifestyle phenotype, rather than as a direct measurement of any single inflammatory mediator. Biomarker values were winsorized at the 1st and 99th percentiles, inverse-normal transformed, and standardized to place all biomarkers on a common scale. The MRS was developed by regressing standardized CII (mean 0, standard deviation [SD] 1) on the 249 standardized biomarkers using elastic net regularized regression, following the metabolomic-signature approach established in prior cohort applications (29, 30). Ten-fold cross-validation was used to select the tuning parameter. The MRS was calculated as the weighted sum of biomarkers with non-zero coefficients and then standardized before validation and mediation analyses.

### 2.6 Statistical analysis

Missing covariate values were imputed using chained equations (31). Covariate missingness patterns and imputation diagnostics are shown in **Supplementary Figures 1** and **S2**. Baseline characteristics were summarized as means with standard deviations for continuous variables and counts with percentages for categorical variables. Kruskal-Wallis tests were used for continuous across-group comparisons, and chi-square tests were used for categorical comparisons.

Cox proportional-hazards models estimated hazard ratios (HRs) and 95% confidence intervals (CIs) for intermediate and high CII groups relative to low CII (32). Each outcome was modeled with its own follow-up time and event indicator. Three sequential models were fitted. Model 0 adjusted for age and sex. Model 1 additionally adjusted for the remaining sociodemographic factors (ethnicity, Townsend deprivation index, and education) and for lifestyle and anthropometric factors (smoking, alcohol frequency, BMI, and physical activity). Model 2 added the clinical domain, multimorbidity, and was prespecified as the primary model.

The proportional-hazards (PH) assumption was assessed with log-minus-log survival plots and scaled Schoenfeld residuals (33). When non-proportionality was detected for a given outcome, the corresponding HRs were interpreted as average associations over follow-up.

Kaplan-Meier cumulative risks were estimated by CII group at 2, 5, and 10 years. Dose-response relationships were examined with restricted cubic spline models for continuous CII, using a reference value of 2 and knots at 0, 2, and 5 (34). The five CII components were analyzed separately using the same covariate structure, with results summarized for LRTI and pneumonia.

Prespecified subgroup analyses assessed the consistency of the CII–ARI association and potential effect modification across clinically relevant strata. Subgroup variables included age (<60 vs ≥60 years), sex (female vs male), BMI (<30 vs ≥30 kg/m²), smoking status (not current vs current), alcohol intake (non-regular vs regular), physical activity (<600 vs ≥600 MET-min/week), and multimorbidity count (0, 1, or ≥2 conditions). Fully adjusted Cox proportional-hazards models were fitted within each stratum, with the low CII group as reference; when a stratifying variable was included in the primary covariate set, it was omitted from the corresponding within-stratum model. Interaction P values were estimated by comparing nested models with and without a CII group × subgroup product term.

Two sensitivity analyses accompanied the primary Cox models. The first excluded the first 2 years of follow-up to reduce concern that undiagnosed illness or early infection events influenced baseline exposure measurements. The second was limited to participants with complete data for all covariates to assess sensitivity to covariate imputation. E-values were calculated for the main high-versus-low CII contrasts, representing the minimum strength of association that an unmeasured confounder would require with both CII and the outcome to move the point estimate, or the lower confidence limit, to the null (35).

**Figure 2:**
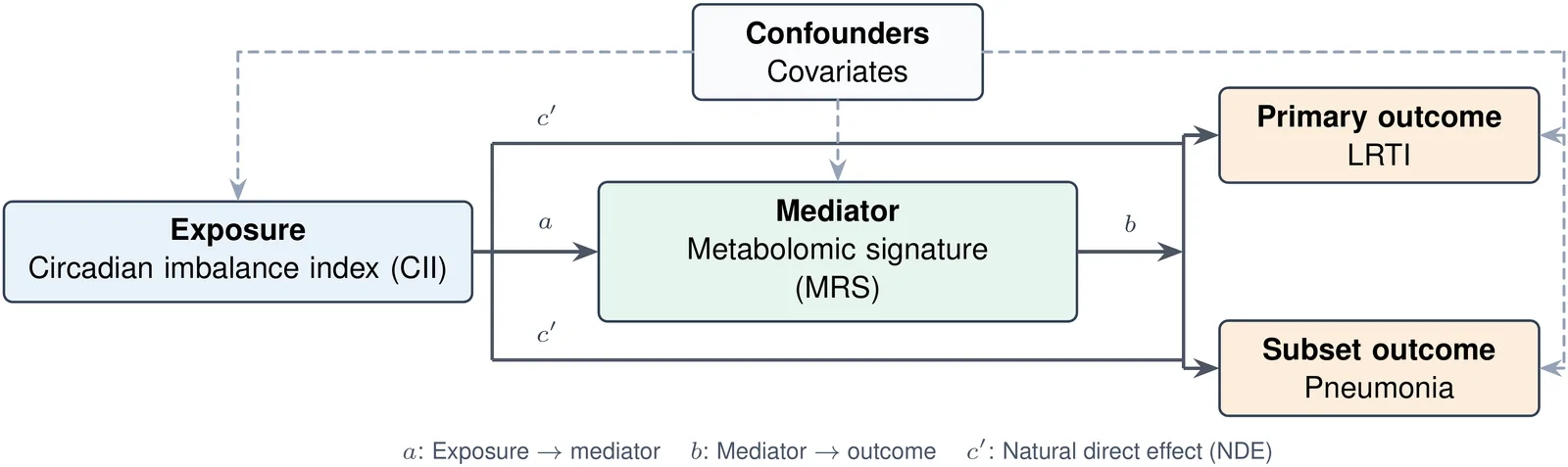
Conceptual mediation framework linking continuous CII, a metabolomic risk signature (MRS), and lower respiratory outcomes. The total effect (TE) is decomposed into the natural direct effect (NDE) and the total natural indirect effect (TNIE) operating through the MRS. CII, Circadian Imbalance Index; LRTI, lower respiratory tract infection; MRS, metabolomic risk signature; NDE, natural direct effect; NMR, nuclear magnetic resonance; TE, total effect; TNIE, total natural indirect effect.

For mediation analyses, LRTI and pneumonia were selected because they were clinically central to the study question and had sufficient event counts for stable metabolomic mediation estimation. Counterfactual mediation models decomposed the total association between standardized continuous CII and each outcome into the natural direct association and the total natural indirect association through the MRS (**Figure 2**) (24, 25). Mediation models used the same prespecified covariate structure as the primary association analyses. Reported estimates include the total effect (TE), natural direct effect (NDE), and total natural indirect effect (TNIE) as HRs, and the proportion mediated (PM) on the log-hazard scale, with 95% CIs and E-values for the indirect association. Biomarker-group decomposition of the MRS was treated as descriptive and reported separately from the primary mediation estimates.

All analyses were performed using R, version 4.3.3 (R Foundation for Statistical Computing, Vienna, Austria). The survival package was used for time-to-event models and PH diagnostics, mice for multiple imputation, rms for restricted cubic splines, glmnet for elastic net regularized regression, CMAverse for counterfactual mediation, and EValue for E-values. Two-sided P values <0.05 were considered statistically significant.

## 3 Results

### 3.1 Participant characteristics

A total of 295,679 participants were included in the analysis (**Figure 1**). According to the CII classification, 103,254 participants (34.9%) were classified as low, 163,969 (55.5%) as intermediate, and 28,456 (9.6%) as high; the score and group distributions are shown in **Supplementary Figure 3**. The mean age was 56.0 years, and 157,311 (53.2%) were female. Baseline characteristics varied across CII groups: the high-CII group exhibited higher BMI, greater socioeconomic deprivation, lower physical activity, higher rates of current smoking, and more multimorbidity compared with the low-CII group (**Table 1**).

**Table 1:** Baseline characteristics according to Circadian Imbalance Index group.

Characteristic	Overall (N = 295,679)	Low CII (n=103,254)	Intermediate CII (n=163,969)	High CII (n=28,456)	P value
Age, years	56.0 (8.1)	57.0 (8.0)	55.7 (8.1)	54.4 (8.1)	<0.001
Female sex	157,311 (53.2)	52,430 (50.8)	88,532 (54.0)	16,349 (57.5)	<0.001
White ethnicity	282,718 (95.6)	101,109 (97.9)	155,259 (94.7)	26,350 (92.6)	<0.001
Education level					<0.001
O levels or less	178,729 (60.4)	60,506 (58.6)	99,713 (60.8)	18,510 (65.0)	
A levels	16,575 (5.6)	5,680 (5.5)	9,126 (5.6)	1,769 (6.2)	
College degree	100,375 (33.9)	37,068 (35.9)	55,130 (33.6)	8,177 (28.7)	
Townsend deprivation index	-1.42 (3.02)	-1.85 (2.76)	-1.30 (3.06)	-0.59 (3.38)	<0.001
BMI, kg/m²	27.4 (4.8)	26.8 (4.2)	27.6 (4.9)	28.5 (5.5)	<0.001
Physical activity, MET-min/week	2678 (2662)	2908 (2715)	2589 (2629)	2353 (2589)	<0.001
Current smoker	30,821 (10.4)	7,405 (7.2)	18,320 (11.2)	5,096 (17.9)	<0.001
Regular alcohol use	131,511 (44.5)	52,051 (50.4)	69,478 (42.4)	9,982 (35.1)	<0.001
Multimorbidity count					<0.001
0	203,934 (69.0)	73,507 (71.2)	112,195 (68.4)	18,232 (64.1)	
1	71,308 (24.1)	23,979 (23.2)	39,890 (24.3)	7,439 (26.1)	
≥2	20,437 (6.9)	5,768 (5.6)	11,884 (7.2)	2,785 (9.8)	

Values are mean (SD) for continuous variables and n (%) for displayed categories of categorical variables. P values compare CII groups and were calculated using Kruskal-Wallis tests for continuous variables and chi-square tests for categorical variables. CII, Circadian Imbalance Index; BMI, body mass index; MET, metabolic equivalent task; SD, standard deviation.

During follow-up, 2,158 URTI events, 21,852 LRTI events, 987 influenza events, 16,320 pneumonia events, and 8,248 other LRTI events were recorded. For pneumonia subtypes, there were 1,942 viral pneumonia events, 960 bacterial pneumonia events, and 14,403 unspecified pneumonia events. Follow-up distributions and event counts by CII group are presented in **Supplementary Figures 4** and **S5**.

### 3.2 Associations between CII and hospital-recorded ARI outcomes

**Figure 3:**
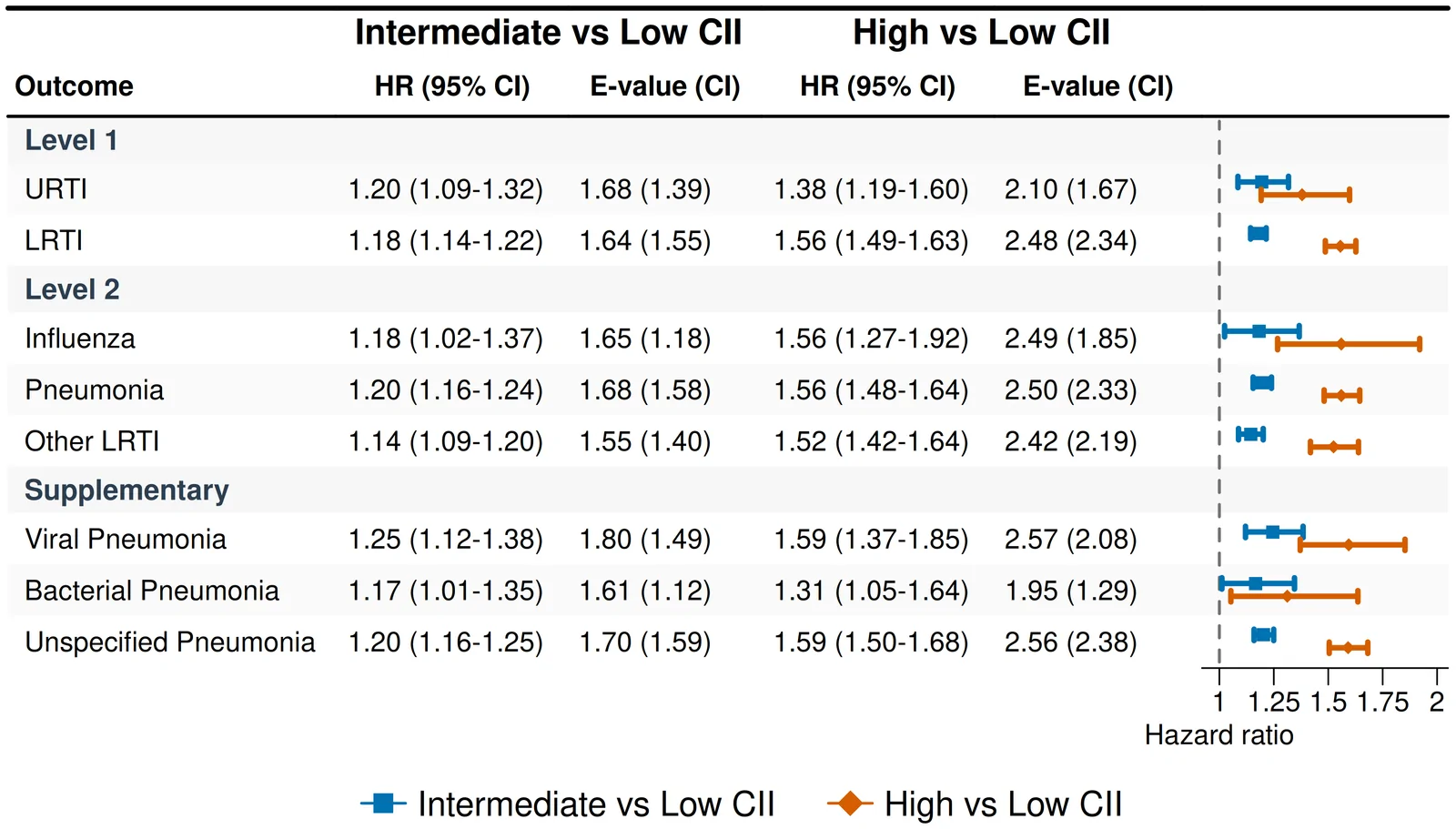
Fully adjusted associations between CII group and incident hospital-recorded acute respiratory infection outcomes. Hazard ratios compare the intermediate and high CII groups with the low CII reference group in Model 2. Points represent HRs, and horizontal lines indicate 95% confidence intervals. E-values quantify the minimum strength of association an unmeasured confounder would require with both CII and the outcome to move the point estimate (or its lower confidence limit) to the null; values are shown as the point-estimate E-value followed in parentheses by the confidence-limit E-value closest to the null. CII, Circadian Imbalance Index; CI, confidence interval; HR, hazard ratio; LRTI, lower respiratory tract infection; URTI, upper respiratory tract infection.

In the fully adjusted model, higher CII demonstrated graded associations with both LRTI and pneumonia (**Figure 3**; **Supplementary Table 1**). For LRTI, compared with the low CII group, the intermediate CII group had an HR of 1.18 (95% CI, 1.14--1.22), and the high CII group had an HR of 1.56 (95% CI, 1.49--1.63). The corresponding E-value for the high versus low CII comparison was 2.48, with a lower confidence-limit E-value of 2.34. For pneumonia, the HR was 1.20 (95% CI, 1.16--1.24) for intermediate CII and 1.56 (95% CI, 1.48--1.64) for high CII; the E-values were 2.50 and 2.33, respectively.

Associations were also positive for the remaining outcomes. High versus low CII was associated with URTI (HR, 1.38; 95% CI, 1.19--1.60), influenza (HR, 1.56; 95% CI, 1.27--1.92), and other LRTI (HR, 1.52; 95% CI, 1.42--1.64). For pneumonia subtypes, positive associations were observed for viral pneumonia (HR, 1.59; 95% CI, 1.37--1.85), bacterial pneumonia (HR, 1.31; 95% CI, 1.05--1.64), and unspecified pneumonia (HR, 1.59; 95% CI, 1.50--1.68). For nearly all outcomes, the estimates for intermediate CII were smaller than those for high CII. PH diagnostics supported the Cox model for most outcomes. Other LRTI was the only outcome with evidence of exposure-specific non-proportionality (P = 0.031), and its HRs should therefore be interpreted as average associations over follow-up (**Supplementary Figures 6**, **S7**).

**Figure 4:**
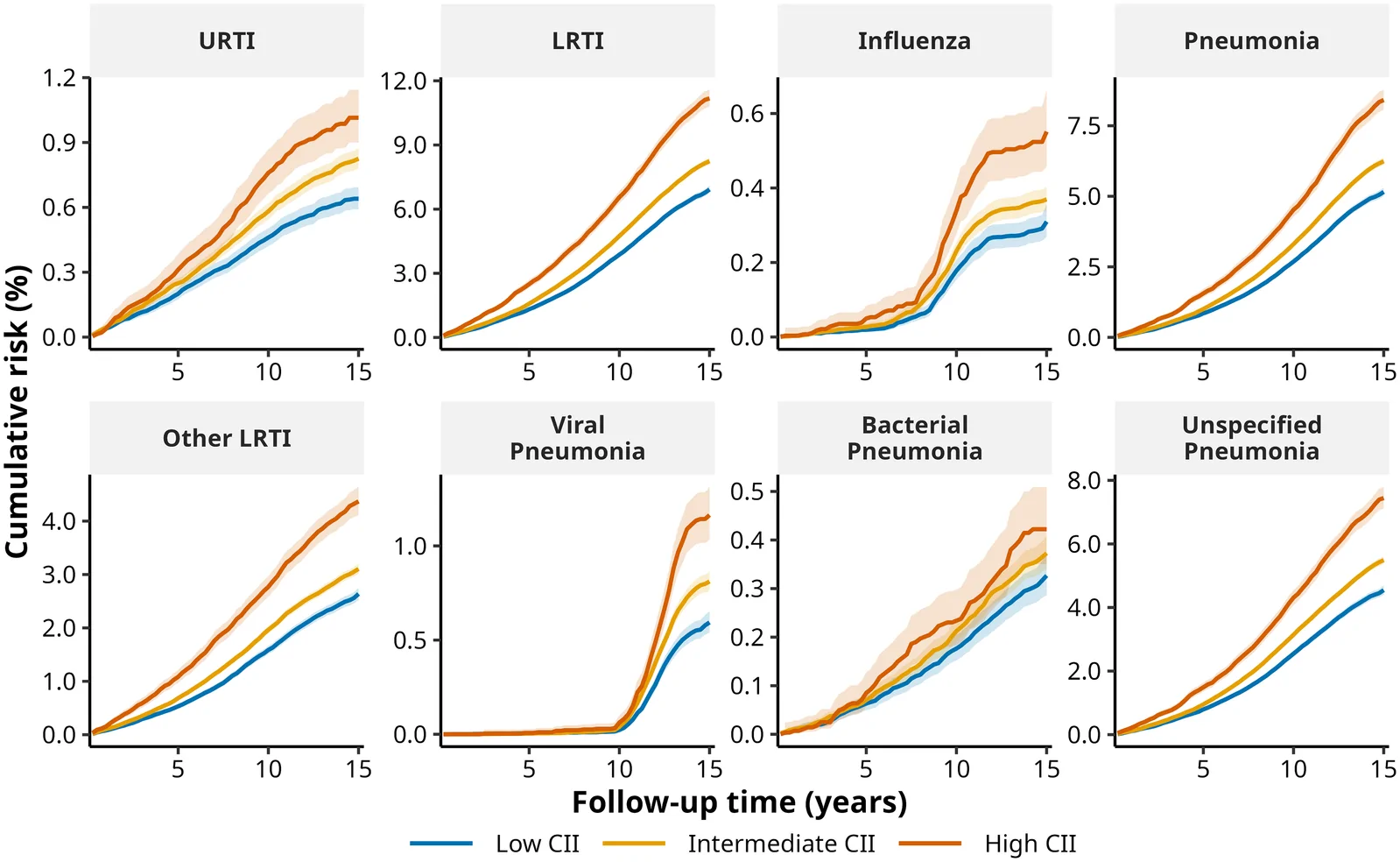
Kaplan-Meier cumulative incidence patterns across hospital-recorded acute respiratory infection outcomes by CII group. CII, Circadian Imbalance Index; LRTI, lower respiratory tract infection; URTI, upper respiratory tract infection.

Cumulative incidence curves demonstrated a graded absolute-risk separation across CII groups (**Figure 4**; **Supplementary Table 2**). For LRTI, the 10-year Kaplan-Meier estimate increased from 3.87% (95% CI, 3.76%--3.99%) in the low-CII group to 4.74% (95% CI, 4.64%--4.85%) in the intermediate group and 6.56% (95% CI, 6.27%--6.85%) in the high group. For pneumonia, the corresponding estimates were 2.69% (95% CI, 2.59%--2.79%), 3.30% (95% CI, 3.22%--3.39%), and 4.52% (95% CI, 4.29%--4.78%). Risk separation was apparent by 2 years, sustained at 5 years, and widened through 10 years.

**Figure 5:**
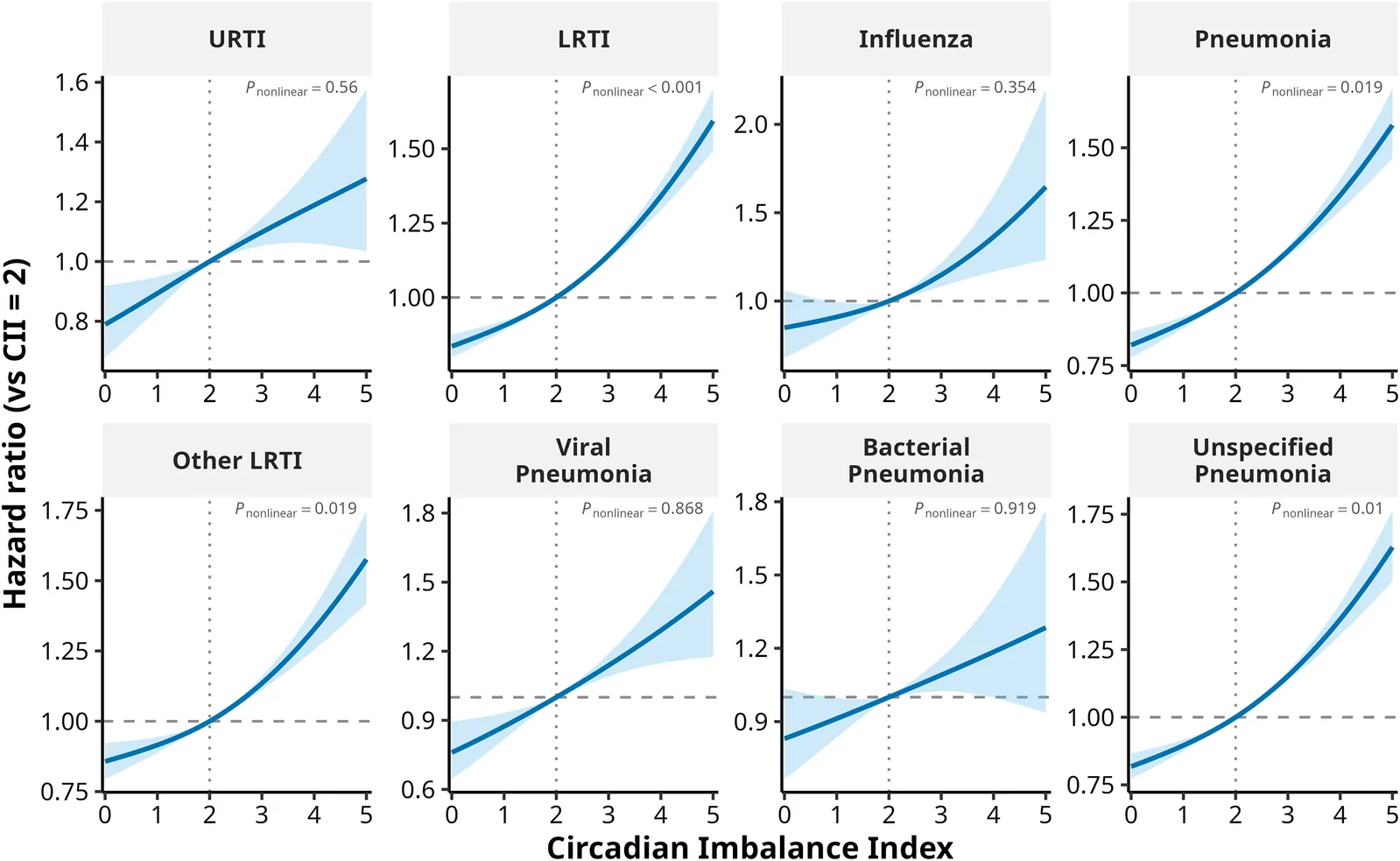
Restricted cubic spline dose-response analyses for standardized continuous CII. Curves show adjusted hazard ratios relative to CII = 2, with 95% confidence bands and outcome-specific tests for overall and nonlinear association. CII, Circadian Imbalance Index; HR, hazard ratio; LRTI, lower respiratory tract infection; URTI, upper respiratory tract infection.

Restricted cubic spline analyses supported a continuous risk gradient across the CII range (**Figure 5**). Overall spline tests were significant for all outcomes. Nonlinearity tests were significant for LRTI, pneumonia, other LRTI, and unspecified pneumonia; however, the plotted curves did not suggest a single abrupt threshold.

### 3.3 Component and subgroup analyses

In the component analyses, each of the five CII components showed positive associations with LRTI and pneumonia (**Supplementary Figure 8**; **Supplementary Table 3**). For LRTI, adjusted HRs ranged from 1.08 (95% CI, 1.05 to 1.11) for evening chronotype to 1.21 (95% CI, 1.17 to 1.24) for atypical sleep duration. Pneumonia estimates were concordant, ranging from 1.07 to 1.21. The larger component estimates were observed for atypical sleep duration, high neuroticism, and low vitamin D.

Across prespecified subgroup strata, the associations between higher CII and hospital-recorded ARI outcomes were generally consistent with the primary analyses (**Supplementary Table 4**). For LRTI, the high-versus-low CII HR was 1.64 (95% CI, 1.54 to 1.75) among women and 1.49 (95% CI, 1.40 to 1.59) among men, with a nominal interaction by sex (P-interaction = 0.005). For pneumonia, the corresponding HRs were 1.66 (95% CI, 1.54 to 1.80) among women and 1.49 (95% CI, 1.39 to 1.60) among men (P-interaction = 0.015). A nominal interaction was also observed for LRTI by age, with high-versus-low CII HRs of 1.55 (95% CI, 1.45 to 1.67) among participants aged <60 years and 1.44 (95% CI, 1.35 to 1.52) among those aged ≥60 years (P-interaction = 0.011). The full subgroup results and interaction pattern are shown in **Supplementary Table 4** and **Supplementary Figure 9**.

### 3.4 Sensitivity analyses

After excluding the first 2 years of follow-up, the high-versus-low CII HR was 1.54 (95% CI, 1.47 to 1.62) for LRTI and 1.54 (95% CI, 1.46 to 1.63) for pneumonia; intermediate CII remained associated with both outcomes (**Supplementary Figure 10**; **Supplementary Table 5**). Among 237,494 complete cases, high-versus-low HRs were 1.52 (95% CI, 1.45 to 1.61) for LRTI and 1.50 (95% CI, 1.41 to 1.60) for pneumonia (**Supplementary Figure 11**; **Supplementary Table 6**).

### 3.5 MRS and mediation analyses

The mediation sample comprised 264,889 participants with complete baseline data for the 249-biomarker NMR panel, including 19,572 LRTI events and 14,614 pneumonia events. The selected MRS biomarkers and coefficients are listed in **Supplementary Table 7**. The 10-fold cross-validation coefficient of determination (R²) was 0.0446, with a Pearson correlation of 0.211. After covariate adjustment, standardized CII was associated with the MRS (beta, 0.1163; standard error [SE], 0.0018; P < 0.001; partial R², 0.0162). These validation metrics indicated that the MRS captured a modest CII-related metabolic signal. The MRS was associated with LRTI (HR, 1.235; 95% CI, 1.216 to 1.255) and pneumonia (HR, 1.244; 95% CI, 1.221 to 1.268) after adjustment for CII and covariates (**Supplementary Table 8**).

In the counterfactual mediation models (**Table 2**), the TE HR per 1-SD higher CII was 1.151 (95% CI, 1.135 to 1.168) for LRTI. The NDE HR was 1.123 (95% CI, 1.107 to 1.140), and the TNIE HR through the MRS was 1.025 (95% CI, 1.023 to 1.027). The PM was 18.5% (95% CI, 16.2% to 20.7%). For pneumonia, the TE HR was 1.152 (95% CI, 1.133 to 1.172). The NDE HR was 1.123 (95% CI, 1.105 to 1.142), the TNIE HR was 1.026 (95% CI, 1.023 to 1.028), and the PM was 19.0% (95% CI, 16.4% to 21.6%). These findings indicate that the NMR-derived MRS, interpreted as a circulating inflammatory load index, captures a quantitatively meaningful portion of the lifestyle-anchored inflammatory burden through which behavioral, dietary, and hormonal exposures translate into respiratory infection susceptibility at the population scale.

**Table 2:** Main MRS mediation analysis for LRTI and pneumonia.

Outcome	Events/N	TE HR (95% CI)	NDE HR (95% CI)	TNIE HR (95% CI)	PM % (95% CI)	E-value
LRTI	19,572/264,889	1.151 (1.135–1.168)	1.123 (1.107–1.140)	1.025 (1.023–1.027)	18.5% (16.2%–20.7%)	1.18 (1.18)
Pneumonia	14,614/264,889	1.152 (1.133–1.172)	1.123 (1.105–1.142)	1.026 (1.023–1.028)	19.0% (16.4%–21.6%)	1.19 (1.18)

Events/N gives the number of outcome events divided by the mediation-analysis sample size. The MRS was developed from 249 standard Nightingale biomarkers measured at baseline by regressing standardized CII on standardized biomarkers with elastic net regularized regression. Mediation estimates use standardized continuous CII; PM is calculated on the log-hazard scale and is not a decomposition of the categorical high-versus-low CII hazard ratio. The number in parentheses after each E-value was calculated from the 95% CI bound closest to HR = 1. TE, total effect; NDE, natural direct effect; TNIE, total natural indirect effect; PM, proportion mediated; MRS, metabolomic risk signature; LRTI, lower respiratory tract infection; HR, hazard ratio; CI, confidence interval.

A descriptive biomarker-group decomposition identified fatty acids as the largest contributing class, with group-level mediated proportions of 10.6% for LRTI and 11.1% for pneumonia (**Supplementary Figure 12**; **Supplementary Table 9**). Inflammation, fluid balance, and relative lipoprotein lipid concentrations contributed smaller indirect associations.

## 4 Discussion

In this large prospective cohort from the UK Biobank, higher CII was associated with an increased risk of incident hospital-recorded ARIs. The associations were graded across CII categories, evident on both relative and absolute scales, and remained similar after excluding early follow-up years and in complete-case analyses. LRTI and pneumonia were the primary outcomes in the metabolomic mediation analysis; for these endpoints, the Nightingale NMR-derived MRS accounted for approximately one-fifth of the association with continuous CII. These findings support a lifestyle-driven low-grade inflammation framework for susceptibility to hospital-recorded ARIs. Prior cohort evidence also links inflammatory status measured before infection with later pneumonia risk, including baseline inflammatory proteins in the Framingham Offspring cohort and CRP-based markers in long-term Finnish data (36, 37). The present study extends this evidence from individual inflammatory markers to an integrated lifestyle-anchored phenotype and its circulating MRS.

The operationalization of CII as an integrative lifestyle-anchored inflammatory phenotype aligns with growing recognition that chronic low-grade systemic inflammation arises not from a single behavioral exposure but from the convergence of sleep, psychosocial, dietary, and hormonal factors (1, 2). Each of these dimensions has independently been linked to inflammatory dysregulation: sleep disruption alters cytokine homeostasis and innate immune function (8, 9), chronic psychosocial stress induces glucocorticoid receptor desensitization and pro-inflammatory transcriptional programs (11, 12), dietary patterns shape circulating fatty-acid composition and metabolic-inflammatory cross-talk (2, 19), and hormonal status, particularly vitamin D, modulates innate antimicrobial responses through Toll-like-receptor-induced cathelicidin expression (15). The graded association observed across CII categories, together with the NMR-derived MRS dominated by fatty-acid biomarkers and including GlycA, a well-established readout of low-grade systemic inflammation (22, 23), provides population-scale, biomarker-anchored support for a unified lifestyle–low-grade-inflammation axis that meaningfully translates into ARI susceptibility.

The subgroup analyses showed that the CII–ARI association was broadly retained across clinically relevant strata, while also suggesting stronger associations for LRTI and pneumonia among women. This pattern is biologically plausible, given established sex differences in immune regulation, inflammatory responses, and circadian physiology (38, 39). These findings support the view that lifestyle-anchored inflammatory burden may operate across population subgroups while leaving room for biological heterogeneity in susceptibility to severe respiratory infections.

Several previous studies have examined individual circadian-related features as separate exposures rather than as a cumulative profile. For example, insufficient sleep has been associated with respiratory infection in population-based data (40), sleep-immune crosstalk is supported by experimental and clinical evidence (8, 9), and experimentally measured shorter sleep has been linked to greater susceptibility to the common cold (41, 42). Evidence for other components of the CII is more indirect but still relevant: chronotype has been associated with morbidity patterns in the UK Biobank (43), psychological stress with susceptibility to the common cold and immune dysregulation (10, 11), vitamin D with heterogeneous protection against ARIs in trial syntheses (13, 14), and caffeine intake with circadian phase shifting under controlled conditions (17). The present study extends this literature by evaluating these domains jointly as the CII, examining whether their cumulative profile is associated with future hospital-recorded respiratory infection risk rather than treating each component as an isolated exposure.

The CII brings together several domains with plausible links to immune regulation and respiratory host defense. Circadian clock research provides one anchor: daily rhythms organize leukocyte trafficking, cytokine release, epithelial barrier function, antimicrobial responses, and lung inflammatory responses to viral challenge (3–7). Other CII components add related but distinct biology. Sleep loss can shift inflammatory homeostasis and weaken host defense (8, 9), and experimental cold-challenge studies link shorter sleep with infection susceptibility (41, 42). Stress-related traits may act through neuroendocrine-immune regulation; chronic stress has been associated with glucocorticoid receptor resistance and a stronger inflammatory response after rhinovirus exposure (10–12). Vitamin D signaling can support innate antimicrobial responses through Toll-like receptor-induced pathways and cathelicidin expression (15), while trial meta-analyses show possible but heterogeneous protection against ARIs (13, 14, 16). Caffeine-related behavior is more indirect, but controlled human data show that caffeine can delay human circadian phase (17). Together, these lines of evidence support a convergence model in which CII marks several immune-relevant domains that may meet at immune timing, inflammatory control, and respiratory host defense.

The mediation findings can be interpreted through the same immunometabolic lens. Immune activation depends on coordinated changes in glycolysis, amino acid handling, fatty acid metabolism, and lipid remodeling; systemic metabolism is therefore not separate from host defense, as broadly framed by immunometabolism research linking metabolic programs to host-pathogen interactions (19, 44). Large-scale Nightingale NMR studies have shown that baseline metabolic profiles can predict future susceptibility to severe pneumonia and coronavirus disease 2019 (COVID-19), and that GlycA captures systemic inflammation and risk of severe infection (21–23). Against this background, the CII-related MRS is best understood as a compact NMR summary of the metabolic state accompanying higher CII. In the mediation analysis, the MRS explained approximately one-fifth (∼20%) of the association between continuous CII and the risk of LRTI and pneumonia, suggesting that circulating metabolomic alterations account for a meaningful portion of the CII–infection link.

The biomarker group decomposition provides a detailed view of where the MRS signal was concentrated. Fatty acids contributed the largest group-level mediated proportions, consistent with immunometabolic evidence that fatty acid metabolism can shape immune cell function and inflammatory signaling (19). GlycA and related inflammatory glycoproteins have known links to systemic inflammation and severe infection risk (22, 23); however, their smaller contribution here suggests that the MRS was not simply an inflammatory marker score. Contributions from fluid balance and relative lipoprotein lipid measures are more likely to reflect broader metabolic context. This interpretation aligns with earlier Nightingale NMR work demonstrating metabolic susceptibility signatures for severe pneumonia and COVID-19 (21), and further refines the picture by showing that fatty acid metabolism drives the MRS signal linking CII to infection risk.

The disproportionate contribution of fatty-acid-related biomarkers to the mediation effect reinforces an immunometabolic interpretation of the MRS. Fatty-acid metabolism is now recognized as a central regulator of innate immune cell function, including processes such as macrophage polarization, neutrophil activation, and dendritic-cell antigen presentation, and is sensitive to dietary, hormonal, and circadian-related inputs (2, 19). The smaller but non-negligible contribution of GlycA and other glycoprotein-acetylation signals further suggests that the MRS is not a single-pathway inflammatory marker but a composite immunometabolic readout, integrating fatty-acid remodeling, low-grade inflammation, and broader systemic metabolic context (22, 23). This composite nature is consistent with the conceptual framing of the present Research Topic, which treats lifestyle-driven inflammation as a multidimensional and biomarker-quantifiable continuum rather than a single-cytokine endophenotype.

The lifestyle-anchored inflammatory phenotype identified in this study may also be interpreted within the broader concept of inflammaging, the age-related accumulation of low-grade systemic inflammation that accompanies declining homeostatic and antimicrobial capacity (45). Although the present analysis does not directly assess age-related inflammatory accumulation over time, the graded associations of CII with respiratory infection in this middle-aged and older UK Biobank population are compatible with the hypothesis that lifestyle-anchored inflammatory burden contributes to the inflammatory environment underlying age-related immune decline and increased infection susceptibility.

The study has several strengths. The large, prospective UK Biobank cohort, with long-term follow-up and linkage to hospital inpatient records, enabled outcome-specific evaluation of hospital-recorded respiratory infections. By using CII as a composite profile, the analysis assessed whether multiple lifestyle-anchored inflammatory domains collectively carry information about infection risk, rather than re-testing each factor in isolation. The MRS provided a composite circulating biomarker signature that may reflect lifestyle-related immunometabolic disturbances, and the mediation analysis showed that the MRS explained about 20% of the CII effect, supporting an immunometabolic interpretation of the observed associations.

Several limitations should be considered. CII was derived from baseline UK Biobank variables rather than direct circadian phenotyping, and several of its components were self-reported; therefore, changes in sleep, behavior, vitamin D status, or caffeine use during follow-up could not be assessed. Hospital inpatient records capture infections requiring hospital care but miss milder episodes managed in the community. Residual confounding remains possible, and the healthy-volunteer profile of the UK Biobank may limit generalizability (46). Traditional acute-phase inflammatory cytokines such as IL-6, CRP, or TNF-alpha were not directly measured; the MRS therefore serves as an NMR-anchored circulating proxy of the low-grade inflammatory and immunometabolic state, rather than as a direct quantification of inflammatory mediators. Future work integrating high-sensitivity inflammatory cytokine panels, mucosal-immune readouts, and longitudinal lifestyle reassessment will further refine the inflammatory load index concept and its mechanistic interpretation.

## 5 Conclusion

In a population-scale prospective cohort, a lifestyle-anchored inflammatory phenotype (the CII) showed graded associations with hospital-recorded ARIs, and a circulating NMR-derived MRS, dominated by fatty-acid-related biomarkers and including the established low-grade inflammation marker GlycA, accounted for approximately one-fifth of the association with LRTI and pneumonia. These findings position lifestyle-driven low-grade inflammation as a biomarker-quantifiable continuum linking modifiable behavioral, dietary, psychosocial, and hormonal exposures to hospital-recorded ARI susceptibility, provide population-scale support for the immunometabolic framework, and may guide future mechanistic studies of immunometabolic pathways connecting lifestyle-related inflammatory burden with host susceptibility.

## Data Availability

The data analyzed in this study is subject to the following licenses/restrictions: Due to UK Biobank data-use agreements, the authors are not permitted to publicly share individual-level participant data. Requests to access these datasets should be directed to UK Biobank Access Management System: https://www.ukbiobank.ac.uk/enable-your-research/apply-for-access.
