# Supplementary material for: A lifestyle-driven inflammatory load index and its circulating metabolomic signature identify susceptibility to hospital-recorded acute respiratory infections: a population-based cohort study of 295,679 adults
Source: Front Endocrinol (Lausanne). 2026 Aug 10;17:1878728. doi: 10.3389/fendo.2026.1878728 (PMC13500250; doi:10.3389/fendo.2026.1878728)
Supplement: Supplementary file 1 [file DataSheet1.docx]

# 1 Supplementary Figures

This file contains Supplementary Figures S1-S12.

**Supplementary Figure S1.** Missingness overview for adjustment covariates after cohort assembly. Bars show the number and percentage of participants with missing values before multiple imputation; CII components and outcome definitions were complete by design after exclusions. BMI, body mass index; CII, Circadian Imbalance Index.

**Supplementary Figure S2.** Imputation diagnostic plot for the first main imputed dataset. Observed and imputed distributions are compared for covariates with missing values to support the MICE workflow. BMI, body mass index; MICE, multiple imputation by chained equations.

**Supplementary Figure S3.** Distribution of the CII score and CII groups in the analytic cohort. Panel A shows the distribution of the summed CII score, and panel B shows the number and percentage of participants in the low, intermediate, and high CII groups. CII, Circadian Imbalance Index.

**Supplementary Figure S4.** Follow-up time distributions among participants with hospital-recorded acute respiratory infection events. Ridge plots summarize outcome-specific event-time distributions across the prespecified outcomes. LRTI, lower respiratory tract infection; URTI, upper respiratory tract infection.

**Supplementary Figure S5.** Event counts by CII group across hospital-recorded acute respiratory infection outcomes. Bars show hospital-recorded events for each outcome within low, intermediate, and high CII groups. CII, Circadian Imbalance Index; LRTI, lower respiratory tract infection; URTI, upper respiratory tract infection.

**Supplementary Figure S6.** Unadjusted log-minus-log survival curves across CII groups. Curves provide a visual check of proportional-hazards behavior for the CII-group exposure across hospital-recorded acute respiratory infection outcomes. CII, Circadian Imbalance Index; LRTI, lower respiratory tract infection; PH, proportional hazards; URTI, upper respiratory tract infection.

**Supplementary Figure S7.** Schoenfeld residual diagnostics for CII-group terms in fully adjusted Cox models. Smoothed residual patterns and outcome-specific PH test P values are shown for the hospital-recorded acute respiratory infection outcomes. CII, Circadian Imbalance Index; PH, proportional hazards; LRTI, lower respiratory tract infection; URTI, upper respiratory tract infection.

**Supplementary Figure S8.** Component-level Cox associations for the five CII components across hospital-recorded acute respiratory infection outcomes. Points and horizontal lines show fully adjusted HRs and 95% CIs for each binary component. CII, Circadian Imbalance Index; CI, confidence interval; HR, hazard ratio; LRTI, lower respiratory tract infection; URTI, upper respiratory tract infection.

**Supplementary Figure S9.** Heatmap of subgroup interaction P values across prespecified strata and hospital-recorded acute respiratory infection outcomes. Cell values are interaction P values, and color intensity represents -log10(P). Interaction P values were estimated from fully adjusted Cox models with CII group by subgroup product terms. Subgroups were defined by age (<60 or ≥60 years), sex (female or male), BMI (<30 or ≥30 kg/m²), smoking status (not current or current), alcohol intake (non-regular or regular), physical activity (<600 or ≥600 MET-min/week), and multimorbidity count (0, 1, or ≥2 conditions). BMI, body mass index; CII, Circadian Imbalance Index; LRTI, lower respiratory tract infection; MET, metabolic equivalent task; URTI, upper respiratory tract infection.

**Supplementary Figure S10.** Sensitivity analysis excluding the first 2 years of follow-up. Fully adjusted HRs compare intermediate and high CII groups with the low CII group after removal of early events. CII, Circadian Imbalance Index; CI, confidence interval; HR, hazard ratio; N, analysis sample size; LRTI, lower respiratory tract infection; URTI, upper respiratory tract infection.

**Supplementary Figure S11.** Complete-case sensitivity analysis. Fully adjusted HRs compare intermediate and high CII groups with the low CII group among participants with complete covariate data. CII, Circadian Imbalance Index; CI, confidence interval; HR, hazard ratio; N, analysis sample size; LRTI, lower respiratory tract infection; URTI, upper respiratory tract infection.

**Supplementary Figure S12.** Forest plot of biomarker-group contributions to the MRS. Points and horizontal bars represent the PM estimates and their 95% CIs for biomarker groups showing robust positive mediation. MRS, metabolomic risk signature; PM, proportion mediated; LRTI, lower respiratory tract infection.
